# Supplementary material for: A simple hydrogel device with flow-through channels to maintain dissipative non-equilibrium phenomena
Source: Commun Chem. 2020 Nov 13;3:168. doi: 10.1038/s42004-020-00420-y (PMC9814359; doi:10.1038/s42004-020-00420-y)
Supplement: Supplementary file 2 — Supplementary Information [file 42004_2020_420_MOESM2_ESM.pdf]

A simple hydrogel device with flow-through channels to maintain dissipative non-equilibrium phenomena

supplementary information

Brigitta Dúzs and István Szalai*

Institute of Chemistry, Eötvös University, Budapest, Hungary

E-mail: szalai.istvan@chem.elte.hu

Supplementary Figures

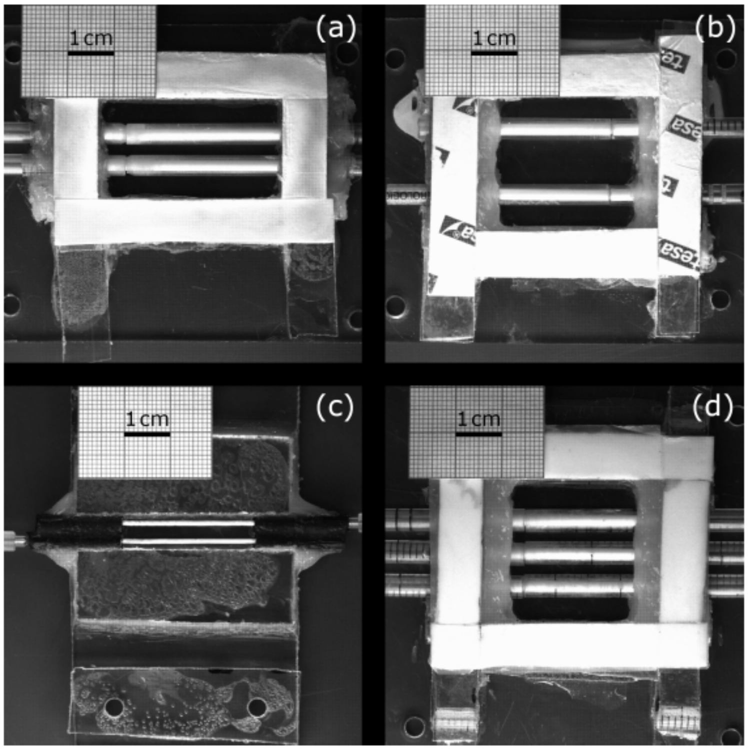

Supplementary Figure 1: Photos of plexi molds used in four different reactor constructions. The devices are different in the diameter of the channels (d), in the distance between the channels (w) and the number of the channels (n). Parameters: (a) d=4.5 mm, w=2.5 mm, n=2; (b) d=4.5 mm, w=10 mm, n=2; (c) d=1.3 mm, w=2.5 mm, n=2; (d) d=4.5 mm, w=2.5 mm, n=3.

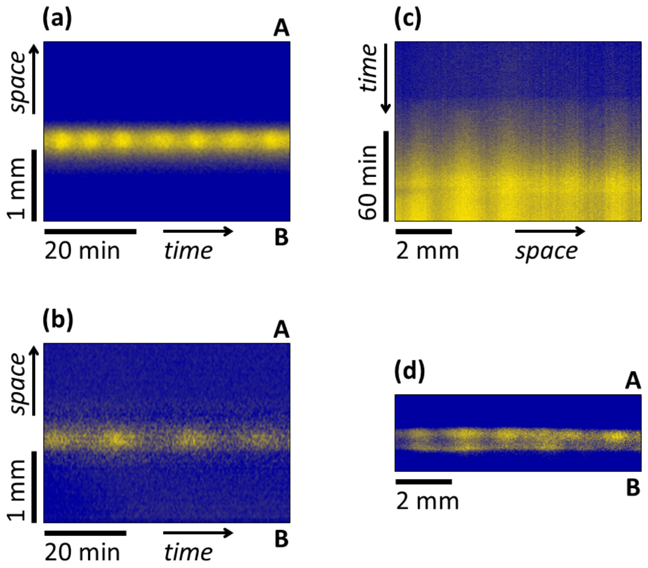

Supplementary Figure 2: Experimental results with the BSF pH oscillator in the miniaturized reactor by using agarose gel (a) and in the normal sized reactor by using polyacrylamide gel (b-d). Space-time plots (a-c) and a snapshot of the stabilized pattern (d). Experimental conditions: (a) $[H_2SO_4]_B$=7 mM, (b) $[H_2SO_4]_B$=6 mM, (c-d) $[H_2SO_4]_B$=10 mM; $[BrO_3^-]_A$=200 mM, $[SO_3^{2-}]_{A,B}$=80 mM, $[Fe(CN)_6^{4-}]_{A,B}$=20 mM, $[BCG]_{A,B}$=0.1 mM, w=2.5 mm, T=35℃.

Supplementary Methods

Derivation of the numerical model

The simulations were made by the dimensionless equations derived from the Rábai model of the pH oscillators (R1)-(R3).[1]

$$A^- + H^+ \rightleftharpoons HA \tag{R1}$$

$$HA + B \xrightarrow{H^+} H^+ + P \tag{R2}$$

$$C + B + H^+ \longrightarrow Q \tag{R3}$$

The corresponding rate equations are the following:

$$v_1 = k_1[\mathrm{A}^-][\mathrm{H}^+] - k_{-1}[\mathrm{HA}] \tag{1}$$

$$v_2 = (k_2[\mathrm{H}^+] + k_2')[\mathrm{HA}][\mathrm{B}] \tag{2}$$

$$v_3 = k_3[\mathrm{B}][\mathrm{C}][\mathrm{H}^+] \tag{3}$$

The dynamics of the gel content is governed by the following set of equations:

$$\partial_t[\mathrm{A}^-] = -k_1[\mathrm{A}^-][\mathrm{H}^+] + k_{-1}[\mathrm{HA}] + D_{\mathrm{A}^-}\Delta[\mathrm{A}^-] \tag{4}$$

$$\partial_t[\mathrm{HA}] = k_1[\mathrm{A}^-][\mathrm{H}^+] - k_{-1}[\mathrm{HA}] - (k_2[\mathrm{H}^+] + k_2')[\mathrm{HA}][\mathrm{B}] + D_{\mathrm{HA}}\Delta[\mathrm{HA}] \tag{5}$$

$$\partial_t[\mathrm{H}^+] = -k_1[\mathrm{A}^-][\mathrm{H}^+] + k_{-1}[\mathrm{HA}] + (k_2[\mathrm{H}^+] + k_2')[\mathrm{HA}][\mathrm{B}] - k_3[\mathrm{B}][\mathrm{C}][\mathrm{H}^+] + D_{\mathrm{H}^+}\Delta[\mathrm{H}^+] \tag{6}$$

$$\partial_t[\mathrm{B}] = -(k_2[\mathrm{H}^+] + k_2')[\mathrm{HA}][\mathrm{B}] - k_3[\mathrm{B}][\mathrm{C}][\mathrm{H}^+] + D_{\mathrm{B}}\Delta[\mathrm{B}] \tag{7}$$

$$\partial_t[\mathrm{C}] = -k_3[\mathrm{B}][\mathrm{C}][\mathrm{H}^+] + D_{\mathrm{C}}\Delta[\mathrm{C}] \tag{8}$$

Here [ ] denotes the space and time dependent concentration in the gel.

Dirichlet boundary conditions were used at the gel/channel surfaces, and no flux boundary conditions were used at the outer surfaces of the gel.

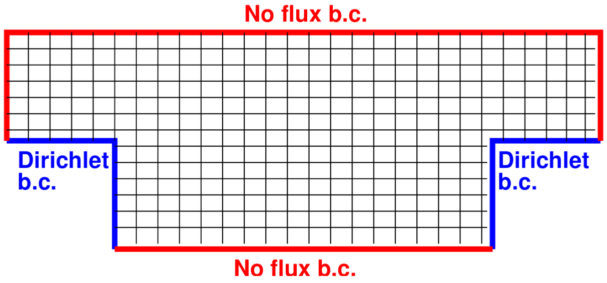

Supplementary Figure 3: The sketch of the applied mesh with boundary conditions

The dimensionless variables are defined as $a = [\mathrm{A}^-]/[\mathrm{A}]_{\mathrm{tot}}$, $a_h = [\mathrm{HA}]/[\mathrm{A}]_{\mathrm{tot}}$, $h = [\mathrm{H}^+]/[\mathrm{A}]_{\mathrm{tot}}$, $b = [\mathrm{B}]/[\mathrm{A}]_{\mathrm{tot}}$, $c = [\mathrm{C}]/[\mathrm{A}]_{\mathrm{tot}}$, where $[\mathrm{A}]_{\mathrm{tot}} = [\mathrm{A}^-] + [\mathrm{HA}]$.

The equations for the content of the gel can be written as:

$$\partial_t a = -\kappa_1 a h + \kappa_{-1} a_h + \Delta a \tag{9}$$

$$\partial_t a_h = \kappa_1 a h - \kappa_{-1} a_h - (\kappa_2 h + \kappa_2') a_h b + \Delta a_h \tag{10}$$

$$\partial_t h = -\kappa_1 a h + \kappa_{-1} a_h + (\kappa_2 h + \kappa_2') a_h b - \kappa_3 b c h + 4\Delta h \tag{11}$$

$$\partial_t b = -(\kappa_2 h + \kappa_2') a_h b - \kappa_3 b c h + \Delta b \tag{12}$$

$$\partial_t c = -\kappa_3 b c h + \Delta c \tag{13}$$

The diffusion coefficients are set to be equal for all species except for the hydrogen ions, which diffuses 4 times faster than the other species.[2]

The parameters are defined as: $\kappa_1 = k_1[\mathrm{A}]_{\mathrm{tot}}/k_0$, $\kappa_{-1} = k_{-1}/k_0$, $\kappa_2 = k_2[\mathrm{A}]_{\mathrm{tot}}^2/k_0$, $\kappa_2' = k_2'[\mathrm{A}]_{\mathrm{tot}}/k_0$, $\kappa_3 = k_3[\mathrm{A}]_{\mathrm{tot}}/k_0$. Here, $k_0 = 2 \times 10^{-3}\,\mathrm{s}^{-1}$ is a reciprocal residence time in the channels. The value of κ_1, κ_{-1}, κ_2, κ_2', κ_3 are set to 5×10^{10}, 5×10^5, 5×10^5, 5×10^1, 5×10^3, respectively. Parameters used in the simulations: $b = 1.5$, $a = 1.0$, $c = 1.0$ in the Left channel and $a = 1.0$, $c = 1.0$ and variable value of h in the Right channel.

Supplementary References

(1) Rabai, G. Modeling and Designing of pH-Controlled Bistability, Oscillations, and Chaos in a Continuous-Flow Stirred Tank Reactor. *ACH - Models Chem.* **1998**, *135*, 381–392.

(2) Schuszter, G.; Gehér-Herczegh, T.; Szűcs, Á.; Tóth, Á.; Horváth, D. Determination of the diffusion coefficient of hydrogen ion in hydrogels. *Phys. Chem. Chem. Phys.* **2017**, *19*, 12136–12143.
