# Supplementary material for: A simple hydrogel device with flow-through channels to maintain dissipative non-equilibrium phenomena
Source: Commun Chem. 2020 Nov 13;3:168. doi: 10.1038/s42004-020-00420-y (PMC9814359; doi:10.1038/s42004-020-00420-y)
Supplement: Supplementary file 3 — Description of Additional Supplementary Files [file 42004_2020_420_MOESM3_ESM.pdf]

Description of Additional Supplementary Files

Name: Supplementary Movie 1
Description: Assembly of the reactor and gel preparation

Name: Supplementary Movie 2
Description: Stripe formation in the reaction between calcium ions and carbonate ions

Name: Supplementary Movie 3
Description: Wave phenomenon in the bromate-sulfite-ferrocyanide reaction

Name: Supplementary Movie 4
Description: Wave phenomenon in the chlorite-iodide-malonic acid reaction

Name: Supplementary Movie 5
Description: Wave phenomenon in the reaction between aluminum ions and hydroxide ions

Name: Supplementary Movie 6
Description: Turing patterns in the chlorite-iodide-malonic acid reaction

Name: Supplementary Movie 7
Description: Dynamics of the three channels ABA configuration

Name: Supplementary Movie 8
Description: Dynamics of the three channels BAB configuration: two parallel lines of waves

Name: Supplementary Movie 9
Description: Dynamics of the three channels BAB configuration: acidic stripe and waves
